# Association between nocturnal and morning blood pressure in patients with obstructive sleep apnea analysis: according to sex

**DOI:** 10.3389/fcvm.2026.1845145

**Published:** 2026-06-25

**Authors:** Yifei Fang, Peng Zhang, Xiao Wang, Chan Sun, Zhenran Chen, Fumei Shang, Yi Miao, Qingzhu Lu, Yuanyuan Ren, Boyue Ma, Haixia Gong, Mengyang Jing, Sonyyun Ouyang, Dawei Zheng

**Affiliations:** 1 Department of Respiratory and Critical Care Medicine, Nanyang Central Hospital, Nanyang, China; 2 Department of Medical Oncology, Nanyang Central Hospital, Nanyang, China; 3 Department of Respiratory and Sleep Medicine, The First Affiliated Hospital of Zhengzhou University, Zhengzhou, China

**Keywords:** blood pressure, evening blood pressure, hypertension, morning blood pressure, obstructive sleep apnea, sex

## Abstract

**Background:**

Previous study focused on the relationship between morning blood pressure (BP) and OSA severity, and the effect of sex on hypertension in OSA. This study was undertaken to investigate the potential effect of evening systolic BP (SBP) and diastolic BP (DBP) on morning SBP and DBP for different genders in OSA patients.

**Methods:**

We conducted a retrospective study of 911 patients diagnosed with OSA via polysomnography (PSG). The association between evening and morning blood pressure was evaluated using multivariable-adjusted logistic regression models and restricted cubic splines (RCS) to assess linearity and dose-response relationships. All analyses were stratified by sex.

**Results:**

Of the 911 participants, 766 (84.08%) were male and 145 (15.92%) were female. Multivariable-adjusted analyses revealed a consistent positive association between evening and morning BP in both sexes. However, subgroup analyses identified distinct effect modifiers: in males, the association was intensified by diabetes mellitus, whereas in females, it was most evident among those with non-severe OSA. Furthermore, severe OSA significantly exacerbated morning BP surges in males with high evening BP, a synergistic effect notably absent in females.

**Conclusion:**

Evening SBP and DBP exhibited a linear association with morning BP across both sexes. However, this relationship was subject to significant effect modification: in males, diabetes mellitus intensified the association, whereas in females, it was most evident among those with non-severe OSA. Consequently, elevated evening BP precipitated marked morning BP surges in males, a phenomenon notably absent in females.

## Introduction

Obstructive sleep apnea (OSA) is a prevalent respiratory disorder characterized by recurrent upper airway collapse during sleep, leading to intermittent hypoxia and arousals (1). These pathophysiological disturbances drive sympathetic activation, oxidative stress, and endothelial dysfunction, thereby substantially elevating the risk of cardiovascular diseases, particularly hypertension (HT) (2). Indeed, epidemiological data indicate that 46%–53% of patients with moderate-to-severe OSA suffer from HT, while OSA is present in 30%–50% of hypertensive patients (3, 4).

Under normal physiological conditions, blood pressure (BP) exhibits a nocturnal “dipping” phenomenon. However, this nocturnal dip is often blunted or reversed in OSA patients, correlating closely with disease severity (5–7). Previous investigations have primarily focused on morning BP, reporting inconsistent associations with OSA severity and gender. For instance, some studies found that morning diastolic BP (DBP) was elevated in males with OSA (8, 9), while others noted this association in non-hypertensive cohorts (10). Despite these efforts, the role of evening BP—a potential precursor to morning surges—remains poorly characterized. Furthermore, the extent to which sex modifies this relationship is unclear, as evidence regarding gender disparities in OSA-related HT ranges from higher vulnerability in men (8, 9, 11–14) to no association (15, 16) or even a higher risk in women (17).

Therefore, a critical gap remains regarding how evening BP influences morning BP across different clinical phenotypes of OSA. While the link between OSA and morning hypertension is established, the specific interaction between evening BP, sex, and disease severity requires clarification. To address this, we hypothesized that evening SBP and DBP would exhibit a linear association with morning BP in OSA patients, but this association would be differentially modified by sex, diabetes mellitus, and OSA severity. Accordingly, this study aimed to evaluate the association between evening and morning BP in a large cohort of OSA patients, with a specific focus on sex-specific effect modification.

## Methods

### Study population

**Figure 1:**
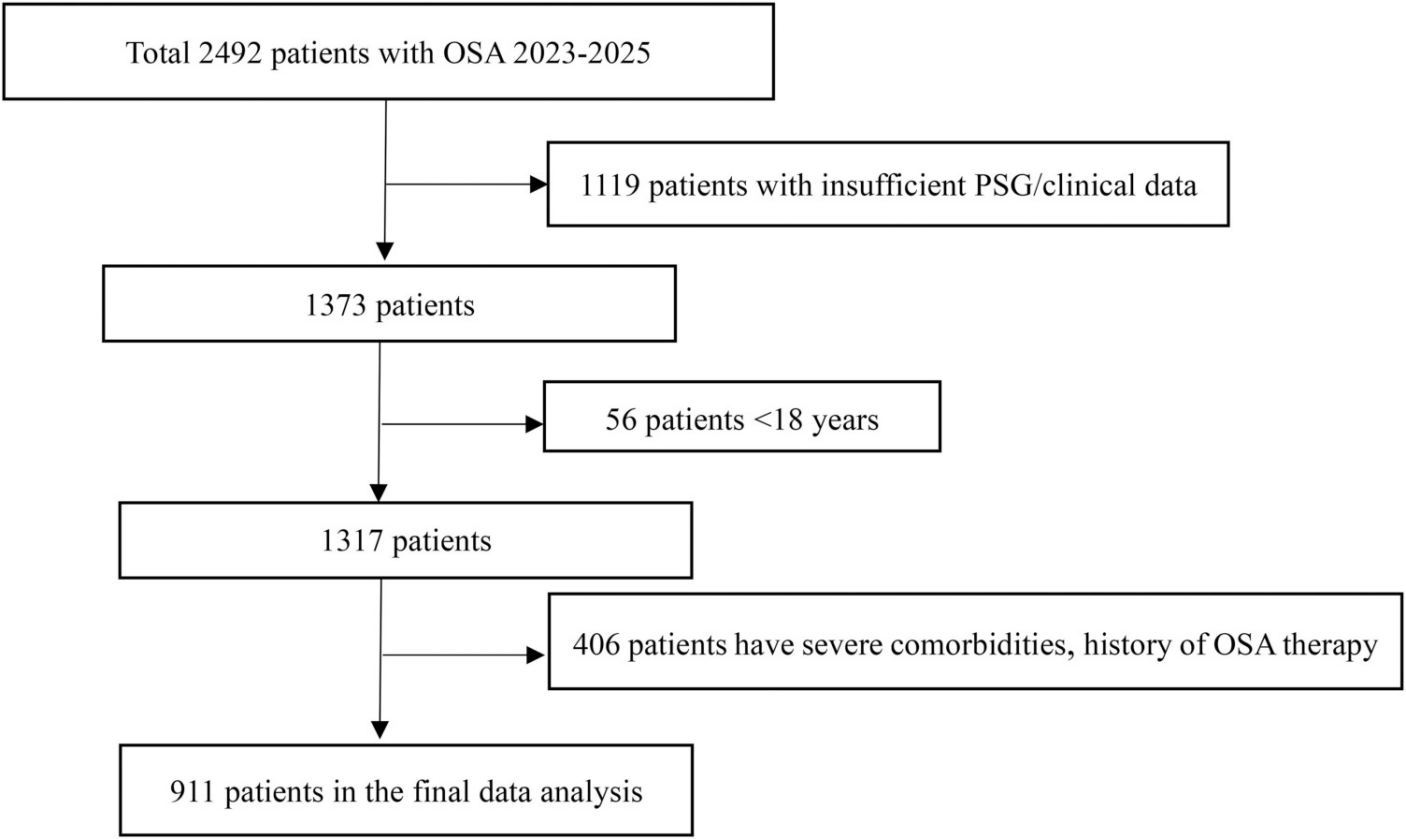
Flow chart of the study.

We retrospectively examined 2,492 subjects diagnosed with OSA between January 2023 and December 2025, and approved by the Ethics Committee of the First Afffliated Hospital of Zhengzhou University (2023-KY-0963–002) and the Ethics Committee of the Central Hospital of Nanyang (20230621052). Dates of patients were collected from the medical record system in a retrospective manner, including sex, age, body mass index (BMI), smoking, alcohol drinking, history of hypertension, diabetes mellitus (DM), coronary artery disease and arrhythmia, sleep parameters and blood pressure (BP). 911 subjects remained after the following exclusion criteria were applied: aged <18 years, insufficient polysomnographic/clinical data, history of OSA therapy, severe heart failure, liver or kidney disease, malignant tumor (Figure 1) (10, 18, 19).

As this is a retrospective study, we were unable to obtain detailed and accurate information regarding the use of antihypertensive and other cardiovascular medications. Notably, approximately 44% of the studied population had hypertension, 11.8% had coronary artery disease, and 12.4% had arrhythmias. Consequently, the medications used to manage these comorbidities may have influenced the reported blood pressure patterns (9, 10, 14, 19).

### Blood pressure measurement protocol

BP was measured in two conditions: in the evening before PSG and in the morning after PSG. During all measurements, patients were awake, with all PSG monitoring equipment attached. Following a 5 min rest period, seated patients underwent three measurements at 5-minute intervals, with mean values subsequently calculated. All BP measurements were performed once on the randomly chosen arm of a sitting patient with sphygmomanometer (Riester, Jungingen, Germany) (9, 10).

According to the BP measured before sleep onset, participants were divided into a high evening BP group and a normal evening BP group. High BP was defined as SBP ≥ 140 mmHg or DBP ≥ 90 mmHg, according to the recommendations of the European Society of Hypertension/European Society of Cardiology guidelines (20).

### Polysomnography

Full-night PSG was carried out at the sleep center in The First Affiliated Hospital of Zhengzhou University and the Central Hospital of Nanyang. Scoring sleep and related events according to the guidelines of the American Academy of Sleep Medicine (AASM) (21). Apnea was defined as a ≥90% drop in flow for at least 10 s. Hypopnea was defined as a 30% or greater drop in breathing amplitude lasting for more than 10 s, accompanied by greater than 3% decrease in oxyhemoglobin saturation or an arousal. Apnea-hypopnea index (AHI) was used to measure the average number of apneas and hypopneas per hour of sleep. Mild, moderate, and severe OSA were defined as AHI of 5–15, 16–30, and >30 events/h, respectively. Mild and moderate OSA were collectively called non-severe OSA. Oxygen Desaturation Index (ODI) was defined as at least 3% decrease in saturation per hour.

### Statistical analyses

The mean ± SD or the median (25th percentile, 75th percentile) and number (%) were used to describe continuous data and categorical data, respectively. For comparison, Student's *t* test, nonparametric Mann–Whitney test and the chi-squared test were used. Univariate linear regression was used to evaluate the associations between morning BP and evening BP. Logistic regression analyses were conducted separately for males and females to evaluate the association of evening SBP and DBP with morning SBP and DBP. Results are presented as odds ratios (ORs) with 95% confidence intervals (CIs), adjusted for relevant covariates. Evening SBP and DBP were assessed by establishing models for both continuous data and categorical data using SBP stratification (140 mmHg ≤ SBP <160 mmHg, 160 mmHg ≤ SBP <180 mmHg, SBP ≥ 180 mmHg) and DBP stratification (90 mmHg ≤ DBP < 100 mmHg, 100 mmHg ≤ DBP < 110 mmHg, DBP ≥ 110 mmHg) with SBP <140 mmHg and DBP <90 mmHg as reference group, respectively. Tests for linear trends across increasing blood pressure strata were performed by modeling the SBP and DBP categories as continuous variables. To characterize the shape of the association between morning and evening blood pressures in OSA patients, we applied restricted cubic splines (RCS) to generate smooth fitting curves. Finally, potential effect modifiers were assessed through prespecified subgroup analyses and formal tests for interaction (22–24). All the data analyses were performed using R version 3.6.1 (http://www.r-project.org) and EmpowerStates (www. empowerstats.com). A 2-sided *P* < 0.05 was considered statistically significant.

## Results

### Baseline characteristics

Table 1 compares the clinical characteristics between the male and female groups and high and normal evening BP groups in each sex. A total of 911 patients were included in the study. There are 766 males, 360 patients with high evening BP and 406 patients with normal evening BP. 59 patients had high evening BP and 86 patients had normal evening BP in 145 females. Compared with female subjects, male subjects were younger, and had higher DBP, more alcohol drinking, more smoking and more severe OSA. AHI was significantly higher in the high evening BP group than in the normal evening BP group (*P* = 0.021) in males, not in females (*P* = 0.278). In male subjects, ODI, minimum SaO_2_, severe OSA and TST were higher in the high evening BP group than in the normal evening BP group. In female subjects, the presence of DM was higher in high evening BP group than in the normal evening BP group. BMI and the presence of HT were higher in high evening BP group than in the normal evening BP group for all genders.

**Table 1:** Characteristics of study participants.

	Total			Male			Female		
Variables	Male (*n* = 766)	Female (*n* = 145)	*P* value	Normal evening BP (*n* = 406)	High evening BP (*n* = 360)	*P* value	Normal evening BP (*n* = 86)	High evening BP (*n* = 59)	*P* value
Age, year	45.07 ± 12.20	54.88 ± 9.81	<0.001***	45.96 ± 12.71	44.06 ± 11.53	0.031*	53.08 ± 10.14	57.51 ± 8.73	0.007**
BMI, kg/m^2^	28.66 ± 3.84	28.28 ± 4.90	0.305	28.10 ± 3.68	29.28 ± 3.92	<0.001***	27.22 ± 4.98	29.83 ± 4.39	0.001**
TST, min	422.75 ± 78.01	426.15 ± 80.70	0.633	414.34 ± 79.22	432.25 ± 75.61	0.001**	420.37 ± 75.49	434.57 ± 87.73	0.300
AHI	38.55 (21.53–57.20)	29.00 (15.30–52.30)	0.012*	35.90 (19.00–56.10)	39.90 (27.70–57.68)	0.021*	25.55 (13.73–49.53)	35.00 (17.35–57.25)	0.278
ODI	41.45 (23.23–59.15)	30.80 (16.10–56.30)	0.018*	38.70 (21.35–57.80)	43.40 (24.78–60.80)	0.038*	27.55 (13.55–55.98)	39.90 (21.90–56.45)	0.332
Mean SaO_2_	91.55 ± 4.22	91.41 ± 5.85	0.745	91.81 ± 3.93	91.25 ± 4.52	0.072	91.65 ± 4.51	91.07 ± 7.42	0.562
Minimum SaO_2_	72.15 ± 12.27	73.10 ± 14.52	0.408	73.30 ± 11.90	70.85 ± 12.56	0.006**	74.04 ± 14.96	71.73 ± 13.86	0.348
Evening SBP, mm Hg	135.09 ± 16.79	135.21 ± 18.62	0.939	123.50 ± 9.92	148.15 ± 12.91	<0.001***	123.35 ± 10.38	152.49 ± 13.81	<0.001***
Evening DBP, mm Hg	84.42 ± 12.74	81.48 ± 11.18	0.010*	76.27 ± 7.80	93.62 ± 10.83	<0.001***	76.41 ± 7.72	88.88 ± 11.36	<0.001***
Morning SBP, mm Hg	135.41 ± 16.52	134.99 ± 17.29	0.778	128.02 ± 13.50	143.75 ± 15.63	<0.001***	127.40 ± 13.71	146.05 ± 16.02	<0.001***
Morning DBP, mm Hg	87.01 ± 12.74	83.54 ± 12.36	0.003**	81.49 ± 10.41	93.24 ± 12.27	<0.001***	79.80 ± 10.20	88.98 ± 13.26	<0.001***
Severe OSA, *n* (%)			<0.001***			<0.001***			0.475
Yes	515 (67.23%)	71 (48.97%)		251 (61.82%)	264 (73.33%)		40 (46.51%)	31 (52.54%)	
No	251 (32.77%)	74 (51.03%)		155 (38.18%)	96 (26.67%)		46 (53.49%)	28 (47.46%)	
Hypertension, *n* (%)			0.068			<0.001***			<0.001***
Yes	328 (42.82%)	74 (51.03%)		124 (30.54%)	204 (56.67%)		24 (27.91%)	50 (84.75%)	
No	438 (57.18%)	71 (48.97%)		282 (69.46%)	156 (43.33%)		62 (72.09%)	9 (15.25%)	
Alcohol drinking, *n* (%)			<0.001***			0.453			–
Yes	264 (34.47%)	0 (0.00%)		135 (33.25%)	129 (35.83%)		0 (0.00%)	0 (0.00%)	
No	502 (65.54%)	145 (100.00%)		271 (66.75%)	231 (64.17%)		86 (100.00%)	59 (100.00%)	
Smoking, *n* (%)			<0.001***			0.441			0.147
Yes	325 (42.43%)	3 (2.07%)		167 (41.13%)	158 (43.89%)		3 (3.49%)	0 (0.00%)	
No	441 (57.57%)	142 (97.93%)		239 (58.87%)	202 (56.11%)		83 (96.51%)	59 (100.00%)	
Diabetes mellitus, *n* (%)			0.015*			0.617			<0.001***
Yes	91 (11.88%)	28 (19.31%)		46 (11.33%)	45 (12.50%)		8 (9.30%)	20 (33.90%)	
No	675 (88.12%)	117 (80.69%)		360 (88.67%)	315 (87.50%)		78 (90.70%)	39 (66.10%)	
Coronary artery disease, *n* (%)			0.006**			0.058			0.191
Yes	81 (10.57%)	27 (18.62%)		51 (12.56%)	30 (8.33%)		13 (15.12%)	14 (23.73%)	
No	685 (89.43%)	118 (81.38%)		355 (87.44%)	330 (91.67%)		73 (84.88%)	45 (76.27%)	
Arrhythmia, *n* (%)			0.168			0.132			0.092
Yes	90 (11.75%)	23 (15.86%)		41 (10.10%)	49 (13.61%)		10 (11.63%)	13 (22.03%)	
No	676 (88.25%)	122 (84.14%)		365 (89.90%)	311 (86.39%)		76 (88.37%)	46 (77.97%)	

BMI, body mass index; TST, total sleep time; AHI, apnea hypopnea index; ODI, oxygen desaturation index; SaO_2_, oxygen saturation; BP, blood pressure; SBP, systolic blood pressure; DBP, diastolic blood pressure.

*** = *P* < 0.001; ** = *P* < 0.01; * = *P* < 0.05.

### Univariate analysis

The results of univariate analysis were shown in Table 2. The results of univariate analysis showed that BMI, evening SBP, evening DBP, AHI, ODI, severe OSA and HT were correlated with higher morning SBP and DBP, whereas minimum SaO_2_ and mean SaO_2_ were negatively associated with higher morning SBP and DBP.

**Table 2:** The results of univariate analysis.

		Morning SBP		Morning DBP	
Variables	Statistics	Effect size (β)	*P* value	Effect size (β)	*P* value
Age	46.63 ± 12.38	0.12 (0.03, 0.20)	0.008**	−0.11 (−0.17, −0.04)	0.002**
BMI	28.60 ± 4.02	1.16 (0.90, 1.42)	<0.001***	0.92 (0.72, 1.12)	<0.001***
TST	423.29 ± 78.41	0.00 (−0.01, 0.02)	0.700	0.01 (−0.00, 0.02)	0.136
AHI	39.37 ± 22.17	0.17 (0.12, 0.22)	<0.001***	0.15 (0.12, 0.19)	<0.001***
ODI	41.49 ± 23.90	0.20 (0.16, 0.25)	<0.001***	0.15 (0.11, 0.18)	<0.001***
Evening SBP	135.11 ± 17.08	0.56 (0.51, 0.61)	<0.001***	0.36 (0.32, 0.41)	<0.001***
Evening DBP	83.95 ± 12.55	0.63 (0.55, 0.70)	<0.001***	0.62 (0.57, 0.67)	<0.001***
Mean SaO_2_	91.53 ± 4.52	−0.58 (−0.82, −0.34)	<0.001***	−0.47 (−0.66, −0.29)	<0.001***
Minimum SaO_2_	72.30 ± 12.65	−0.25 (−0.34, −0.17)	<0.001***	−0.23 (−0.29, −0.16)	<0.001***
Sex					
Male	766 (84.08%)	ref		ref	
Female	145 (15.92%)	−0.43 (−3.38, 2.53)	0.778	−3.47 (−5.73, −1.22)	0.003**
Severe OSA					
Yes	586 (64.32%)	6.26 (4.04, 8.48)	<0.001***	6.13 (4.45, 7.81)	<0.001***
No	325 (35.68%)	ref		ref	
Hypertension					
Yes	402 (44.13%)	11.58 (9.53, 13.62)	<0.001***	7.29 (5.69, 8.89)	<0.001***
No	509 (55.87%)	ref		ref	
Smoking					
Yes	328 (36.00%)	0.23 (−2.03, 2.48)	0.845	0.11 (−1.62, 1.83)	0.903
No	583 (64.00%)	ref		ref	
Alcohol drinking					
Yes	264 (28.98%)	1.19 (−1.19, 3.57)	0.327	1.47 (−0.35, 3.29)	0.114
No	647 (71.02%)	ref		ref	
Diabetes mellitus					
Yes	119 (13.06%)	2.64 (−0.56, 5.84)	0.107	1.51 (−0.94, 3.97)	0.228
No	792 (86.94%)	ref		ref	
Coronary artery disease					
Yes	108 (11.86%)	1.56 (−1.78, 4.91)	0.359	−2.42 (−4.98, 0.13)	0.064
No	803 (88.14%)	ref		ref	
Arrhythmia					
Yes	113 (12.40%)	3.80 (0.53, 7.07)	0.023*	−0.15 (−2.66, 2.36)	0.907
No	798 (87.60%)	ref		ref	

BMI, body mass index; TST, total sleep time; AHI, apnea hypopnea index; ODI, oxygen desaturation index; SaO_2_, oxygen saturation; SBP, systolic blood pressure; DBP, diastolic blood pressure.

*** = *P* < 0.001; ** = *P* < 0.01.

### Evening BP and morning BP in OSA

In male subjects, evening SBP and evening DBP showed positive correlation with morning SBP (β = 0.568, 95% CI: 0.511–0.625, *P* < 0.001; β = 0.619, 95% CI: 0.539–0.700, *P* < 0.001) and morning DBP (β = 0.391, 95% CI: 0.345–0.437, *P* < 0.001; β = 0.610, 95% CI: 0.554–0.666, *P* < 0.001) in crude model (Table 3). In model 1, the result did not have obvious changes. For sensitivity analysis, we also assessed evening BP as categorical variables (SBP < 140 mmHg, 140 mmHg ≤ SBP <160 mmHg, 160 mmHg ≤ SBP <180 mmHg, SBP ≥ 180 mmHg and DBP < 90 mmHg, 90 mmHg ≤ DBP <100 mmHg, 100 mmHg ≤ DBP < 110 mmHg, DBP ≥ 110 mmHg), and found that the same trend was observed as well (*P* for trend < 0.05).

**Table 3:** Relationship between evening BP and morning BP in different models of males.

	Morning SBP				Morning DBP			
Variables	Crude		Model 1		Crude		Model 1	
	β (95% CI)	*P* value	β (95% CI)	*P* value	β (95% CI)	*P* value	β (95% CI)	*P* value
Evening SBP	0.57 (0.51, 0.63)	<0.001***	0.49 (0.43, 0.55)	<0.001***	0.39 (0.35, 0.44)	<0.001***	0.32 (0.27, 0.37)	<0.001***
Evening SBP subgroup								
<140 mmHg	Ref		Ref		Ref		Ref	
≥140, <160 mmHg	14.55 (12.38, 16.72)	<0.001***	12.34 (10.19, 14.48)	<0.001***	9.68 (7.93, 11.43)	<0.001***	7.51 (5.77, 9.25)	<0.001***
≥160, <180 mmHg	27.66 (23.32, 31.99)	<0.001***	23.73 (19.44, 28.02)	<0.001***	18.54 (15.04, 22.04)	<0.001***	14.67 (11.19, 18.15)	<0.001***
≥180 mmHg	28.09 (17.61, 38.56)	<0.001***	23.60 (13.58, 33.63)	<0.001***	20.48 (12.03, 28.93)	<0.001***	16.47 (8.34, 24.60)	<0.001***
*P* for trend		<0.001***		<0.001***		<0.001***		<0.001***
Evening DBP	0.62 (0.54, 0.70)	<0.001***	0.51 (0.42, 0.59)	<0.001***	0.61 (0.55, 0.67)	<0.001***	0.53 (0.47, 0.59)	<0.001***
Evening DBP subgroup								
<90 mmHg	Ref		Ref		Ref		Ref	
≥90, <100 mmHg	10.84 (8.21, 13.46)	<0.001***	9.23 (6.76, 11.75)	<0.001***	10.18 (8.29, 12.06)	<0.001***	8.85 (7.02, 10.69)	<0.001***
≥100, <110 mmHg	16.44 (12.47, 20.42)	<0.001***	11.84 (7.94, 15.75)	<0.001***	17.38 (14.53, 20.23)	<0.001***	14.03 (11.18, 16.88)	<0.001***
≥110 mmHg	25.99 (20.09, 31.90)	<0.001***	21.00 (15.24, 26.77)	<0.001***	24.63 (20.40, 28.86)	<0.001***	21.40 (17.19, 25.61)	<0.001***
*P* for trend		<0.001***		<0.001***		<0.001***		<0.001***

Model 1 was adjusted for age, BMI, hypertension, diabetes mellitus, coronary artery disease, arrhythmia.

*** = *P* < 0.001.

**Figure 2:**
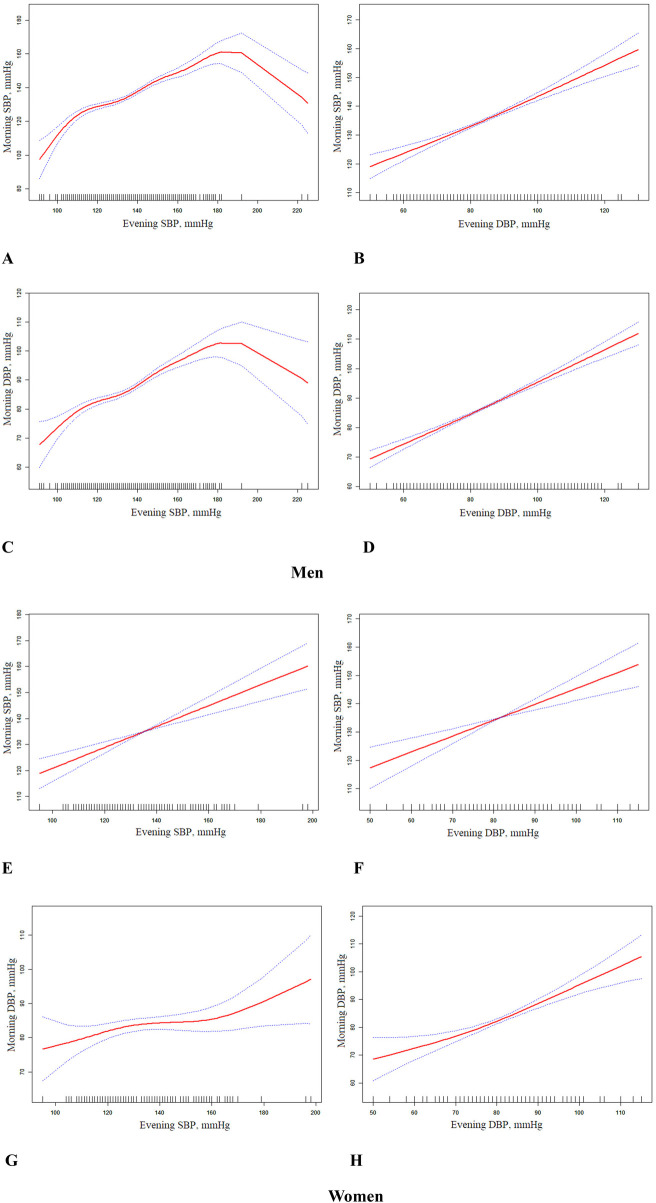
The association between the evening BP and morning BP in men and women with OSA. (**A**) The association between the morning SBP and evening SBP in men. (**B**) The association between the morning SBP and evening DBP in men. (**C**) The association between the morning DBP and evening SBP in men. (**D**) The association between the morning DBP and evening DBP in men. (**E**) The association between the morning SBP and evening SBP in women. (**F**) The association between the morning SBP and evening DBP in women. (**G**) The association between the morning DBP and evening SBP in women. (**H**) The association between the morning DBP and evening DBP in women. The solid line and dashed line represent the estimated values and their corresponding 95% confidence interval, respectively. The adjustment factors included age, BMI, hypertension, diabetes mellitus, coronary artery disease, arrhythmia.

In female subjects, evening SBP and evening DBP also showed positive correlation with morning SBP (β = 0.537, 95% CI: 0.412–0.661, *P* < 0.001; β = 0.718, 95% CI: 0.494–0.943, *P* < 0.001) and morning DBP (β = 0.244, 95% CI:0.143–0.345, *P* < 0.001; β = 0.677, 95% CI: 0.534–0.820, *P* < 0.001) in crude model and model 1 (Table 4). However, when we use evening BP as categorical variables, the same trend can also be observed, except between evening SBP and morning DBP (*P* for trend = 0.139). Further analyses using restricted cubic spline confirmed the linearly positive association between evening BP and morning BP in OSA (Figure 2).

**Table 4:** Relationship between evening BP and morning BP in different models of females.

	Morning SBP				Morning DBP			
Variables	Crude		Model 1		Crude		Model 1	
	β (95% CI)	*P* value	β (95% CI)	*P* value	β (95% CI)	*P* value	β (95% CI)	*P* value
Evening SBP	0.54 (0.41, 0.66)	<0.001***	0.40 (0.26, 0.54)	<0.001***	0.244 (0.14, 0.35)	<0.001***	0.14 (0.02, 0.25)	0.024*
Evening SBP subgroup								
<140 mmHg	Ref		Ref		Ref		Ref	
≥140, <160 mmHg	16.26 (10.72, 21.79)	<0.001***	11.38 (5.42, 17.34)	<0.001***	6.76 (2.38, 11.13)	0.003**	2.47 (−2.29, 7.22)	0.310
≥160, <180 mmHg	18.25 (9.60, 26.89)	<0.001***	11.07 (1.83, 20.31)	0.02*	6.41 (−0.42, 13.24)	0.068	1.16 (−6.21, 8.54)	0.758
≥180 mmHg	41.71 (20.88, 62.54)	<0.001***	34.82 (14.52, 55.13)	<0.001***	25.76 (9.30, 42.21)	0.003**	18.25 (2.05, 34.45)	0.029*
*P* for trend		<0.001***		<0.001***		<0.001***		0.139
Evening DBP	0.72 (0.49, 0.94)	<0.001***	0.56 (0.33, 0.79)	<0.001***	0.68 (0.53, 0.82)	<0.001***	0.59 (0.43, 0.75)	<0.001***
Evening DBP subgroup								
<90 mmHg	Ref		Ref		Ref		Ref	
≥90, <100 mmHg	16.43 (8.49, 24.37)	<0.001***	11.04 (3.52, 18.55)	0.005**	13.53 (8.34, 18.72)	<0.001***	10.89 (5.61, 16.17)	<0.001***
≥100, <110 mmHg	20.38 (8.17, 32.58)	<0.001***	17.07 (5.71, 28.43)	0.003**	20.64 (12.67, 28.61)	<0.001***	18.64 (10.65, 26.62)	<0.001***
≥110 mmHg	16.38 (−1.97, 34.72)	0.08	7.14 (−10.69, 24.96)	0.434	24.31 (12.33, 36.29)	<0.001***	18.04 (5.51, 30.57)	0.006**
*P* for trend		<0.001***		<0.001***		<0.001***		<0.001***

Model 1 was adjusted for age, BMI, hypertension, diabetes mellitus, coronary artery disease, arrhythmia.

*** = *P* < 0.001; ** = *P* < 0.01; * = *P* < 0.05.

### Subgroup analyses

**Figure 3:**
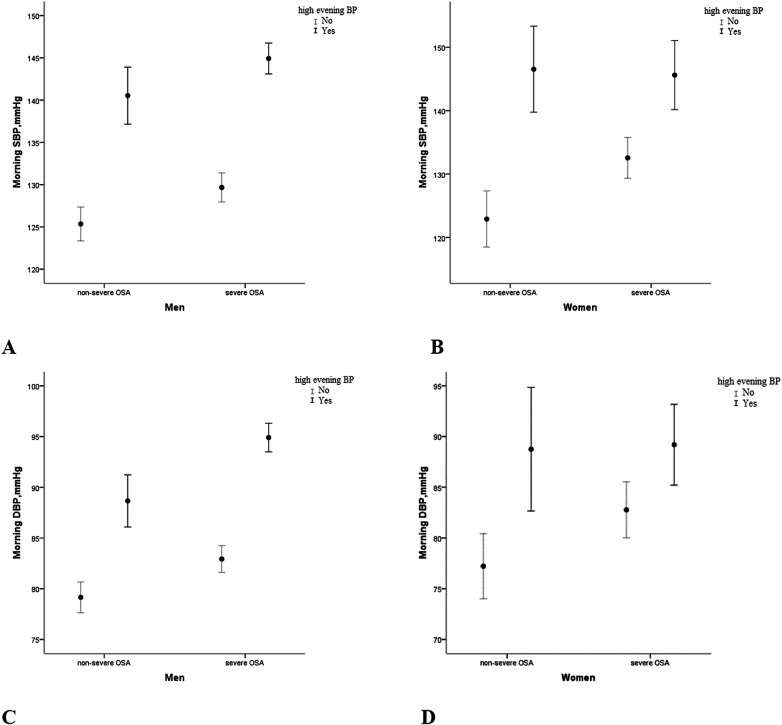
Evening BP with OSA interaction on morning BP in men and women. 95% confidence intervals of morning SBP **(A,B)** and DBP **(C,D)** in subjects with non-severe OSA and severe OSA. Interaction between OSA group and high evening BP was analyzed separately for men and women. BP, blood pressure; OSA, obstructive sleep apnea; SBP, systolic blood pressure; DBP, diastolic blood pressure.

Table 5 shows the results of stratification analysis in male, the test for interactions were significant for DM in evening SBP for morning SBP (*P* for interaction < 0.01) and morning DBP (*P* for interaction = 0.05), while the test for interactions were not statistically significant for age, BMI, smoking, alcohol drinking, severe OSA, hypertension, coronary artery disease, and arrhythmia (all *P*-interaction > 0.05). In each case, the model is not adjusted for the stratification variable. In female subjects (Table 6), the test for interactions were significant for severe OSA in evening SBP and morning DBP for morning SBP (*P* for interaction < 0.001, *P* for interaction = 0.0019) and morning DBP (*P* for interaction = 0.0027, *P* for interaction = 0.00459), while the test for interactions were not statistically significant for age, BMI, hypertension, DM, coronary artery disease and arrhythmia. Figure 3 shows a severe OSA–high evening BP interaction in different gender. There were significant differences in morning SBP and DBP between severe OSA groups with high evening BP [Figure 3 (A,C) in men]. No significant differences in morning SBP and DBP between severe OSA groups with high evening BP were in female [Figure 3 (B,D) in women].

**Table 5:** The association between evening BP and morning BP in various subgroups of males.

Variables		Morning SBP			Morning DBP				Morning SBP			Morning DBP		
	Evening SBP							Evening DBP						
Subgroups	*N*	β (95% CI)	*P* value	*P* for interaction	β (95% CI)	*P* value	*P* for interaction	*N*	β (95% CI)	*P* value	*P* for interaction	β (95% CI)	*P* value	*P* for interaction
Severe OSA				0.114			0.933				0.566			0.218
Yes	515	0.45 (0.38, 0.52)	<0.001***		0.31 (0.25, 0.37)	<0.001***		515	0.48 (0.37, 0.59)	<0.001***		0.55 (0.47, 0.62)	<0.001***	
No	251	0.57 (0.46, 0.67)	<0.001***		0.32 (0.23, 0.40)	<0.001***		251	0.53 (0.38, 0.68)	<0.001***		0.46 (0.35, 0.57)	<0.001***	
Age				0.097			0.651				0.760			0.312
≤40	298	0.52 (0.43, 0.61)	<0.001***		0.32 (0.24, 0.40)	<0.001***		298	0.55 (0.43, 0.68)	<0.001***		0.52 (0.41, 0.62)	<0.001***	
>40, ≤60	375	0.46 (0.38, 0.55)	<0.001***		0.32 (0.25, 0.39)	<0.001***		375	0.53 (0.40, 0.65)	<0.001***		0.53 (0.44, 0.61)	<0.001***	
>60	93	0.61 (0.37, 0.84)	<0.001***		0.35 (0.19, 0.51)	<0.001***		93	0.41 (0.08, 0.73)	0.017*		0.63 (0.44, 0.81)	<0.001***	
BMI				0.151			0.131				0.464			0.314
≤28	360	0.45 (0.36, 0.54)	<0.001***		0.30 (0.22, 0.38)	<0.001***		360	0.49 (0.37, 0.61)	<0.001***		0.52 (0.43, 0.61)	<0.001***	
>28	406	0.55 (0.47, 0.63)	<0.001***		0.37 (0.30, 0.43)	<0.001***		406	0.57 (0.45, 0.69)	<0.001***		0.58 (0.50, 0.66)	<0.001***	
Hypertension				0.574			0.856				0.857			0.311
Yes	328	0.48 (0.39, 0.57)	<0.001***		0.30 (0.23, 0.37)	<0.001***		328	0.53 (0.39, 0.66)	<0.001***		0.56 (0.47, 0.64)	<0.001***	
No	438	0.51 (0.43, 0.59)	<0.001***		0.33 (0.26, 0.40)	<0.001***		438	0.50 (0.39, 0.61)	<0.001***		0.50 (0.42, 0.59)	<0.001***	
Diabetes mellitus				<0.001***			0.050				0.941			0.112
Yes	91	0.54 (0.48, 0.61)	<0.001***		0.34 (0.29, 0.39)	<0.001***		91	0.46 (0.13, 0.78)	0.007**		0.65 (0.45, 0.85)	<0.001***	
No	675	0.19 (0.01, 0.39)	0.071		0.19 (0.05, 0.34)	0.008**		675	0.51 (0.42, 0.60)	<0.001***		0.52 (0.45, 0.58)	<0.001***	
Coronary artery disease				0.549			0.499				0.725			0.374
Yes	81	0.57 (0.37, 0.76)	<0.001***		0.37 (0.22, 0.52)	<0.001***		81	0.48 (0.16, 0.79)	0.004**		0.64 (0.44, 0.83)	<0.001***	
No	685	0.48 (0.42, 0.54)	<0.001***		0.31 (0.26, 0.36)	<0.001***		685	0.51 (0.42, 0.60)	<0.001***		0.52 (0.46, 0.59)	<0.001***	
Arrhythmia				0.134			0.366				0.473			0.798
Yes	90	0.68 (0.53, 0.83)	<0.001***		0.36 (0.24, 0.49)	<0.001***		90	0.61 (0.35, 0.87)	<0.001***		0.56 (0.39, 0.72)	<0.001***	
No	676	0.56 (0.50, 0.63)	<0.001***		0.31 (0.26, 0.36)	<0.001***		676	0.49 (0.40, 0.58)	<0.001***		0.53 (0.47, 0.60)	<0.001***	
Smoking				0.298			0.855				0.728			0.815
Yes	325	0.52 (0.42, 0.62)	<0.001***		0.32 (0.24, 0.40)	<0.001***		325	0.47 (0.33, 0.61)	<0.001***		0.52 (0.42, 0.62)	<0.001***	
No	441	0.47 (0.40, 0.55)	<0.001***		0.32 (0.26, 0.38)	<0.001***		441	0.52 (0.41, 0.63)	<0.001***		0.54 (0.46, 0.62)	<0.001***	
Alcohol drinking				0.271			0.277				0.852			0.535
Yes	264	0.52 (0.41, 0.62)	<0.001***		0.35 (0.26, 0.43)	<0.001***		264	0.46 (0.32, 0.60)	<0.001***		0.54 (0.44, 0.64)	<0.001***	
No	502	0.48 (0.40, 0.55)	<0.001***		0.31 (0.25, 0.36)	<0.001***		502	0.53 (0.42, 0.64)	<0.001***		0.53 (0.45, 0.61)	<0.001***	

Adjusted for: age, BMI, hypertension, diabetes mellitus, coronary artery disease, arrhythmia. In each case, the model is not adjusted for the stratification variable.

*** = *P* < 0.001; ** = *P* < 0.01.

**Table 6:** The association between evening BP and morning BP in various subgroups of females.

Variables		Morning SBP			Morning DBP				Morning SBP			Morning DBP		
	Evening SBP							Evening DBP						
Subgroups	*N*	β (95% CI)	*P* value	*P* for interaction	β (95% CI)	*P* value	*P* for interaction	*N*	β (95% CI)	*P* value	*P* for interaction	β (95% CI)	*P* value	*P* for interaction
Severe OSA				0.0004			0.0027				0.0019			0.0459
Yes	71	0.15 (−0.02, 0.32)	0.085		−0.04 (−0.18, 0.10)	0.564		71	0.20 (−0.08, 0.47)	0.161		0.45 (0.26, 0.64)	<0.001***	
No	74	0.68 (0.47, 0.89)	<0.001***		0.33 (0.15, 0.52)	<0.001***		74	0.92 (0.58, 1.27)	<0.001***		0.75 (0.49, 1.00)	<0.001***	
Age				0.562			0.344				0.759			0.449
≤40	12							12						
>40, ≤60	89	0.34 (0.15, 0.54)	<0.001***		0.16 (−0.01, 0.32)	0.072		89	0.48 (0.14, 0.82)	0.007**		0.71 (0.46, 0.96)	<0.001***	
>60	44	0.44 (0.20, 0.68)	0.001**		0.05 (−0.11, 0.21)	0.555		44	0.65 (0.21, 1.09)	0.007**		0.43 (0.19, 0.68)	0.002**	
BMI				0.521			0.427				0.328			0.438
≤28	79	0.53 (0.31, 0.74)	<0.001***		0.24 (0.08, 0.41)	0.005**		79	0.61 (0.26, 0.95)	<0.001***		0.51 (0.28, 0.75)	<0.001***	
>28	66	0.48 (0.29, 0.67)	<0.001***		0.18 (0.01, 0.35)	0.007**		66	0.49 (0.17, 0.81)	0.004**		0.68 (0.45, 0.90)	<0.001***	
Hypertension				0.696			0.173				0.861			0.417
Yes	74	0.41 (0.22, 0.61)	<0.001***		0.10 (−0.07, 0.26)	0.263		74	0.58 (0.25, 0.91)	0.001**		0.61 (0.38, 0.85)	<0.001***	
No	71	0.35 (0.13, 0.58)	0.003**		0.21 (0.04, 0.37)	0.017*		71	0.44 (0.09, 0.79)	0.015*		0.49 (0.26, 0.72)	<0.001***	
Diabetes mellitus				0.938			0.776				0.839			0.193
Yes	28	0.45 (0.19, 0.71)	0.002**		0.12 (−0.16, 0.39)	0.409		28	0.64 (0.13, 1.16)	0.023*		0.86 (0.51, 1.21)	<0.001***	
No	117	0.39 (0.22, 0.56)	<0.001***		0.13 (−0.00, 0.26)	0.059		117	0.57 (0.31, 0.84)	<0.001***		0.52 (0.34, 0.71)	<0.001***	
Coronary artery disease				0.647			0.876				0.290			0.527
Yes	27	0.59 (0.26, 0.91)	0.002**		0.22 (−0.03, 0.47)	0.097		27	0.31 (−0.30, 0.92)	0.331		0.50 (0.16, 0.83)	0.009**	
No	118	0.36 (0.20, 0.52)	<0.001***		0.13 (−0.01, 0.26)	0.066		118	0.63 (0.38, 0.88)	<0.001***		0.62 (0.43, 0.80)	<0.001***	
Arrhythmia				0.901			0.721				0.645			0.505
Yes	23	0.53 (0.11, 0.95)	0.024*		0.08 (−0.28, 0.44)	0.676		23	0.55 (−0.17, 1.27)	0.151		0.81 (0.41, 1.20)	<0.001***	
No	122	0.39 (0.24, 0.54)	<0.001***		0.15 (0.02, 0.27)	0.025*		122	0.57 (0.33, 0.82)	<0.001***		0.56 (0.38, 0.74)	<0.001***	

Adjusted for: age, BMI, hypertension, diabetes mellitus, coronary artery disease, arrhythmia. In each case, the model is not adjusted for the stratification variable.

*** = *P* < 0.001; ** = *P* < 0.01; * = *P* < 0.05.

## Discussion

This study provides novel insights into the gender-specific association between evening and morning blood pressure in OSA patients. By employing restricted cubic splines and comprehensive interaction analyses, we have confirmed a linear relationship that persists across the full spectrum of hypertension severity. Unlike studies relying on binary categorization, our quantitative approach captures the continuum of risk, underscoring the clinical imperative to monitor evening BP in both male and female OSA patients. This study elucidates the gender-specific effects of evening SBP and DBP on morning BP in OSA patients. In males, adjusted models confirmed robust positive associations between evening and morning BP, validated as linear by restricted cubic splines. This association persisted in participants with DM. In females, while continuous evening BP correlated with morning levels, the link between evening SBP and morning DBP was inconsistent in sensitivity analyses. Notably, severe OSA exacerbated morning BP surges only in males with high evening BP, not in females. Paradoxically, the strongest correlations were observed in participants with non-severe OSA, suggesting a potential decoupling of the hypertensive response at advanced disease stages.

Prior research indicates that OSA primarily exerts its hypertensive effects during nighttime, often blunting the physiological nocturnal BP dip (25). Consistent with this, our multivariable-adjusted analysis revealed a significant dose-response relationship in males, evidenced by incrementally increasing β coefficients across rising evening SBP/DBP categories and a significant *P* for trend < 0.05. This robust linear association was further substantiated by restricted cubic spline analyses. Our findings align with Lavie-Nevo et al., who similarly reported a linear relationship between the evening-to-morning BP surge and OSA severity in men (12), and corroborate the broader epidemiological observation that OSA frequently manifests as isolated nocturnal hypertension (26). However, the interaction with DM introduces a critical nuance. While OSA is mechanistically linked to DM, our stratification analysis revealed that evening SBP—but not DBP—predicted morning BP levels exclusively in participants with DM. This contrasts with the more generalized effect observed in non-diabetic subjects. We attribute this phenomenon to the advanced vascular aging characteristic of diabetic patients. With an older mean age (51.1 ± 11.1 vs. 44.3 ± 12.1 years) and likely higher prevalence of arterial stiffness, the vasculature of OSA patients with DM may be less dynamic. In this context, evening SBP may serve not merely as a marker of nocturnal sympathetic tone, but as a reflection of established arterial rigidity. This distinction is clinically pivotal: while managing OSA is a standard recommendation, our data suggest that for patients with comorbid DM, aggressive glycemic control is paramount to mitigate the fixed vascular risk that contributes to morning BP elevation.

In female subjects, both evening SBP and DBP as continuous variables demonstrated positive correlations with morning BP. However, a nuanced dissociation emerged in the sensitivity analysis: evening SBP was not associated with morning DBP, despite the restricted cubic spline indicating a linear trend. While this apparent discrepancy may partially reflect the limited statistical power inherent in subgroup analyses of female patients, we propose it represents a true physiological phenomenon rather than mere chance. This interpretation is supported by the striking heterogeneity observed across OSA severity. Unlike in males, where severe OSA strongly drove morning BP elevation, we found that only women with non-severe OSA exhibited significant positive correlations between evening and morning BP. This aligns with the epidemiological reality reported by the Wisconsin Sleep Cohort Study, which noted a substantially lower prevalence of moderate-to-severe OSA in women (5.6%) compared to men (13.0%) (27). The skewed distribution in our cohort—where 67.23% of males had severe OSA vs. only 48.97% of females (*P* < 0.05)—suggests that the hemodynamic impact of OSA in women may manifest differently. We hypothesize that the female vasculature is more resilient to the stressors of intermittent hypoxia until a certain threshold is breached. In mild-to-moderate OSA, the vascular system may still be responsive, translating evening pressure surges into morning levels. However, in severe OSA, compensatory mechanisms (potentially estrogen-mediated vasodilation or altered autonomic regulation) may decouple this relationship to protect against extreme surges. Consequently, our findings do not contradict previous literature but rather refine it: clinical vigilance for hypertension should be highest in women with mild-to-moderate OSA, as this is the phenotype where the BP-elevating effect is most consistently expressed.

Multiple mechanisms underpin the relationship between hypertension (HT) and OSA, with intermittent hypoxia (IH) being a pivotal driver (28–30). IH triggers a two-pronged pathological cascade: firstly, by activating the sympathetic nervous system and renin-angiotensin axis via chemoreceptor sensitization and catecholamine surges, leading to vasoconstriction; and secondly, by inducing reactive oxygen species, which promote endothelial dysfunction, inflammation, and arterial remodeling (28–30). Conversely, severe HT itself may exacerbate OSA through fluid shifts and pharyngeal edema, creating a vicious cycle (31–33).

Our findings both corroborate and extend these established mechanisms. We observed that severe OSA with high evening BP significantly impacted morning SBP and DBP, aligning with Cho et al. regarding sleep-trough SBP increases (34) and with studies linking morning DBP to OSA severity (10). However, our data reveal a critical nuance in the differential regulation of systolic vs. diastolic pressure. While SBP elevation is classically attributed to increased conductance vessel resistance and arterial stiffness (35), our results suggest that in the context of OSA, the effect on DBP typically linked to peripheral resistance and sympathetic tone is more complex (8, 10, 36).

Specifically, our research diverges from studies reporting a universal association between DBP and OSA severity across all subjects (9). We propose that this discrepancy is not a contradiction but reflects effect modification by clinical phenotype. For instance, the influence of IH on DBP may be attenuated in patients with advanced arterial stiffness, where the vasculature is less compliant and SBP becomes the dominant marker. Furthermore, the augmented sympathetic activity we observed, likely triggered by pre-morning apneic events (37, 38), may interact with these structural changes differently across sexes and comorbidities. Therefore, the “missing link” in our understanding is not a single unknown mechanism, but rather how these overlapping pathways are weighted differently in specific patient subgroups. Future studies should focus on disentangling these interactions rather than seeking a monolithic explanation.

Our study reveals a profound effect modification by sex on the relationship between evening and morning BP in OSA. While males exhibited robust positive correlations between evening SBP/DBP and morning levels, the association in females was distinctly heterogeneous: evening SBP correlated with morning SBP, yet this link was absent for DBP in sensitivity analyses, and evening DBP predicted both morning SBP and DBP. This sexual dimorphism was further amplified by disease severity. Severe OSA acted as a potent driver of morning BP elevation in men, whereas it exerted no significant effect on morning BP in women. These findings help reconcile the long-standing controversy in the literature. While many studies reported that men are more vulnerable to OSA-related hypertension (8, 9, 11–14), and others find no gender association (15, 16)or even heightened risk in women (17), our data suggest these inconsistencies may stem from differences in phenotypic expression rather than true biological contradictions. We propose that the female vasculature possesses a relative resilience to intermittent hypoxia. Supporting this, experimental evidence demonstrates that intact female rats exposed to hypoxia are less likely to develop hypertension compared to males or ovariectomized females (39). Sex hormones, particularly estrogen, play a pivotal role in modulating adaptive responses to stressors like hypoxia (35, 39), potentially preserving vascular reactivity in pre-menopausal or early postmenopausal women. Consequently, the lack of BP elevation in our female cohort with severe OSA does not negate the pathology of OSA; rather, it suggests a decoupling of the hypertensive response. As evidenced by population data showing blunted nocturnal BP dips in postmenopausal women (40), the female cardiovascular system may adapt differently. Our results indicate that the window for effective intervention might differ by sex: while aggressive management of severe OSA is critical for men to curb morning surges, our data underscore the necessity of monitoring women with mild-to-moderate OSA, where the BP-elevating effect appears most pronounced before potential compensatory mechanisms take hold.

The clinical significance of our findings is underscored by established prognostic thresholds: evening BP ≥ 120/70 mmHg is a potent predictor of adverse cardiovascular outcomes (41–43). Our results corroborate and extend this evidence to the OSA population. By demonstrating a strong, independent effect of evening BP on subsequent morning BP, we provide a mechanistic link for the high cardiovascular risk in these patients. This aligns with previous observations that evening BP fluctuations are driven by nocturnal hypoxia and arousals (44), and supports the notion that the non-dipper/rising BP pattern prevalent in OSA (5–7)may originate from pre-sleep hemodynamic status. Our data offer a fresh perspective on the ongoing controversy regarding the optimal timing of antihypertensive administration (45, 46). While the benefits of nighttime dosing remain debated, our findings suggest that targeting evening BP may be a more precise strategy than simply shifting medication schedules. If evening BP acts as the hemodynamic bridge to morning surges, then strict control of evening BP—rather than just morning or 24 h averages—should be the therapeutic goal, particularly in high-risk groups such as males with severe OSA or patients with comorbid diabetes. However, we acknowledge that the specific features linking OSA to evening BP elevation require further elucidation. Future research should focus on whether interventions aimed specifically at reducing evening BP can disrupt this pathway and improve cardiovascular prognosis. Clarifying these mechanisms is not merely an academic exercise, but a necessary step toward personalized chronotherapeutic strategies for OSA patients.

Several limitations of this study warrant consideration. First, the retrospective design precludes causal inference regarding the relationship between OSA and blood pressure fluctuations, nor can it determine whether the observed high BP group will progress to clinical hypertension over time. Second, the absence of 24 h ambulatory blood pressure monitoring limits our ability to characterize diurnal BP patterns comprehensively. Third, the cohort is predominantly male, which may limit the generalizability of the findings to the female population. Finally, detailed and accurate information regarding the use of antihypertensive and other medications was unavailable. Given that different classes of antihypertensive agents may exert distinct effects on the duration and severity of respiratory events, this represents a potential confounding factor that could not be fully adjusted for.

## Conclusion

Evening SBP and DBP exhibited a linear association with morning BP across both sexes in this OSA cohort. However, the strength of this association was significantly modified by clinical context: diabetes mellitus exacerbated the effect in males, whereas the relationship was most prominent in females with non-severe OSA. Notably, elevated evening BP precipitated significant morning BP surges in males, a finding not replicated in females. These results underscore the presence of sexual dimorphism in the hemodynamic consequences of OSA and highlight the necessity of sex-specific strategies for managing evening BP to prevent morning hypertension.

## Data Availability

The raw data supporting the conclusions of this article will be made available by the authors, without undue reservation.
